# Müllerian-type clear cell adenocarcinoma of the urethra: a case report

**DOI:** 10.31744/einstein_journal/2026RC1169

**Published:** 2026-03-13

**Authors:** Júlia Costa Linhares, Andressa Caroline Martins de Souza, Renata Namie Yoshioka Kimura, Paulo Eduardo Dietrich Jaworski, Ana Paula Martins Sebastião, Samya Hamad Mehanna

**Affiliations:** 1 Universidade Federal do Paraná Curitiba PR Brazil Postgraduate student in the Obstetrics, Gynecology, and Women's Health Program, Universidade Federal do Paraná, Curitiba, PR, Brazil.; 2 Faculdade Evangélica Mackenzie do Paraná Curitiba PR Brazil Faculdade Evangélica Mackenzie do Paraná, Curitiba, PR, Brazil.; 3 Hospital Universitário Evangélico Curitiba PR Brazil Hospital Universitário Evangélico, Curitiba, PR, Brazil.; 4 Faculdade Evangélica Mackenzie do Paraná Curitiba PR Brazil Faculdade Evangélica Mackenzie do Paraná, Curitiba, PR, Brazil.; 5 Universidade Federal do Paraná Faculty Member in the Obstetrics, Gynecology, and Women's Health Program Curitiba PR Brazil Faculty Member in the Obstetrics, Gynecology, and Women's Health Program, Universidade Federal do Paraná, Curitiba, PR, Brazil.

**Keywords:** Urethral neoplasms, Adenocarcinoma, clear cell, Female urogenital diseases, Müllerian ducts

## Abstract

Primary urethral carcinoma is a rare malignancy, particularly in women, that often presents at an advanced stage with nonspecific symptoms. Among the histological subtypes, clear cell adenocarcinoma is exceptionally uncommon, with approximately only 250 cases reported in the English literature, posing diagnostic and management challenges. We report a case of Müllerian-type clear cell adenocarcinoma of the urethra in a 58-year-old woman. Imaging studies excluded gynecological origins, suggesting derivation from a Müllerian duct remnant or metaplasia. Histologically, the tumor displayed the classic features of clear cell carcinoma, which were supported by positive PAX8 and napsin A immunostaining. Primary urethral adenocarcinoma appears to have poor prognosis in women. Current management relies on a multimodal approach that combines surgery, chemotherapy, and radiotherapy and has shown promising outcomes in recent studies. This case highlights the importance of considering clear cell adenocarcinomas in the differential diagnosis of urethral tumors.

## INTRODUCTION

Primary urethral carcinoma is an uncommon malignancy that accounts for less than 1% of all genitourinary tumors. Although it is more frequent in men (male-to-female ratio: 2.9:1), women typically present with more advanced disease and higher cancer-specific mortality rates.^(1)^ The disease mainly affects older adults, particularly those aged 75 years.^(1,2)^

Transitional (urothelial) and squamous cell carcinomas are the predominant histological subtypes, whereas adenocarcinomas represent approximately 16% of the primary urethral neoplasms. Among adenocarcinomas, colonic/mucinous variants are the most common, whereas clear cell variants account for only about 15% of cases.^(3,4)^

Clear cell adenocarcinoma of the urethra, representing approximately 0.003% of female genitourinary malignancies, presents major diagnostic difficulties owing to its rarity and nonspecific symptoms, such as hematuria, dysuria, urinary frequency, obstructive symptoms, pelvic pain, or an extra-urethral mass.^(3,5)^

Owing to its rarity and challenging diagnosis, detailed case descriptions are crucial to improve our understanding of its clinicopathological spectrum and management. Here, we present a case of primary Müllerian-type clear cell adenocarcinoma of the female urethra.

## CASE REPORT

A 58-year-old woman presented with five days of dysuria and hematuria. Her medical history included menopause at 44 years of age, hypertension, diabetes, a history of smoking, vaginal delivery, and sling surgery performed 20 years earlier.

The initial abdominopelvic CT revealed bladder distention without a visible tumor. Cystoscopy revealed a vegetative lesion extending along the entire urethra, predominantly on the right lateral wall and roof, with invasion into the bladder neck.

**Figure 1:**
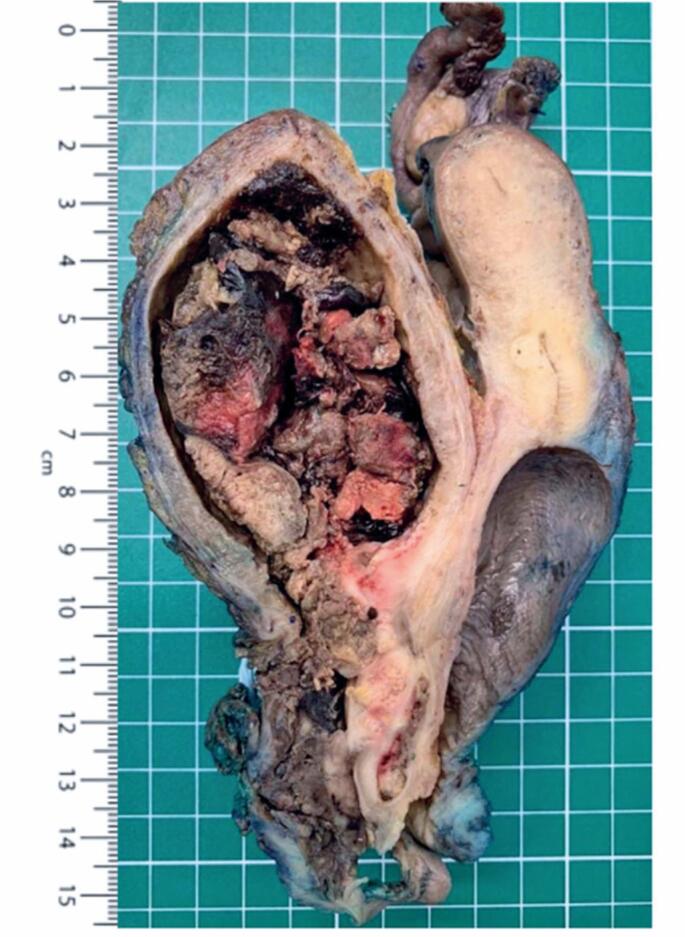
Of anterior pelvic exenteration specimen. Uterus present, bilateral uterine adnexa, bladder, vagina, and vulva en bloc. Note the presence of a large lesion centered on the urethra and projecting into the bladder lumen

**Figure 2:**
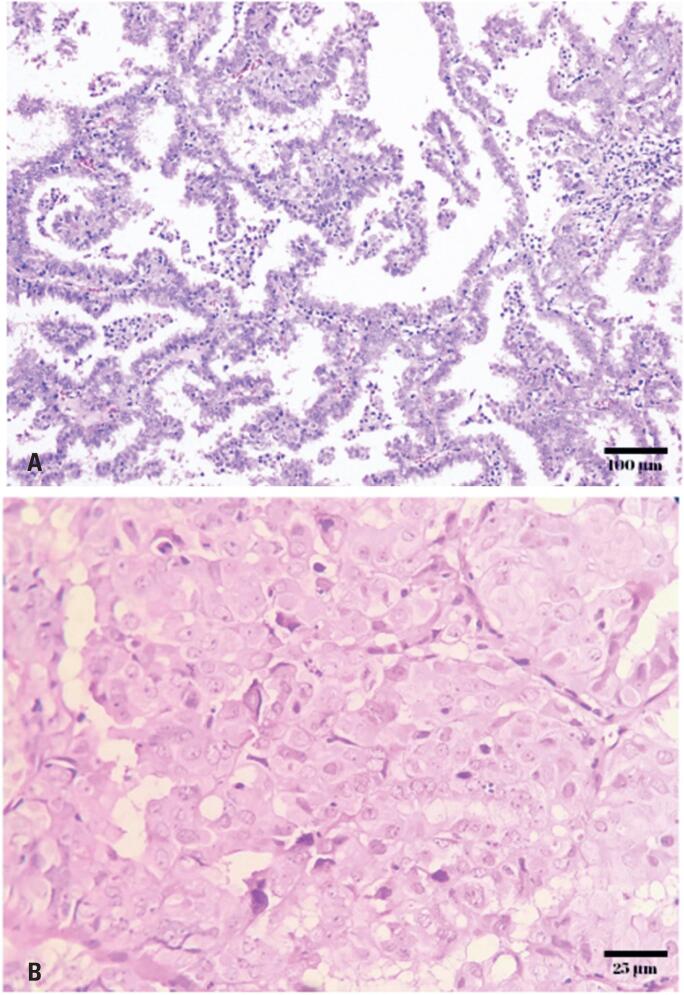
A) Urethral lesion biopsy, invasive papillary adenocarcinoma, H&E, original magnification x100. B) Clear cytoplasm with high-grade nuclei, H&E, original magnification x400

Transvaginal ultrasonography reveals endometrial thickening. Staging studies have reported no evidence of distant implants. Given the extent of the tumor, anterior pelvic exenteration with complete vaginal closure and urinary reconstruction were performed using the Bricker technique. Intraoperatively, a urethral tumor projecting into the bladder was identified (Figure 1), measuring 11.8 × 6.8 × 4.2cm and infiltrating the uterine serosa. Pathology confirmed a high-grade Müllerian-type clear cell adenocarcinoma (Figure 2). Pathological staging, according to the AJCC on Cancer 8th edition/Union for International Cancer Control system for urethral carcinoma, revealed pT4 pN2 pM1 with three of the nine lymph nodes involved.

**Figure 3:**
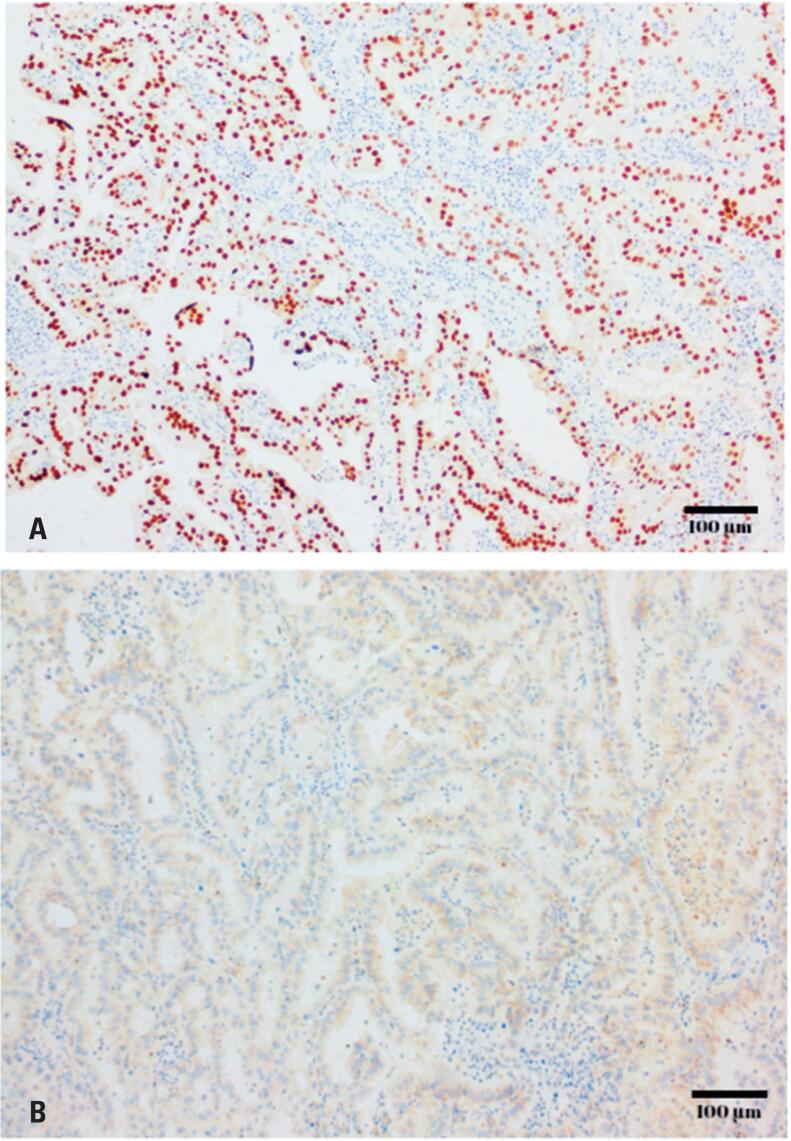
Immunohistochemical profile of the urethral tumor. A) Diffuse nuclear PAX8 positivity (immunohistochemistry with hematoxylin counterstain, original magnification x100). B) Absence of GATA3 expression (immunohistochemistry with hematoxylin counterstain, original magnification x100)

Immunohistochemistry revealed diffuse nuclear PAX8 (Figure 3A) and cytoplasmic napsin A positivity consistent with Müllerian differentiation. The tumor was CK7-positive and negative for CK20, p63, p40, GATA3 (Figure 3B), ER, PR, TTF-1, and racemase (AMACR). Although additional markers such as HNF-1β, PAX2, ARID1A, and mismatch-repair proteins were unavailable at our institution, the overall morphology and immunoprofile supported a diagnosis of Müllerian-type clear cell adenocarcinoma, effectively excluding urothelial and renal origins.

One month after surgery, imaging revealed multiple pulmonary nodules. The patient was started on carboplatin plus paclitaxel; however, after six cycles, massive pleural effusion occurred, necessitating a switch to gemcitabine. The patient subsequently progressed to cachexia and died eight months after surgery.

This study was approved by the Research Ethics Committee of *Faculdade Evangélica Mackenzie do Paraná* (CAAE 34258220.0.0000.0103; #4.166.910).

## DISCUSSION

Primary urethral tumors are rare, particularly in women, and often present aggressively or at advanced stages.^(1,3)^ Most are epithelial in origin and include squamous cell carcinoma, urothelial carcinoma, and adenocarcinoma. The adenocarcinoma group included colonic/mucinous, cribriform, and clear-cell (Müllerian) variants.^(1,4)^

Clear cell adenocarcinoma occurs predominantly in women aged approximately 58 years and accounts for approximately 0.003% of female genitourinary malignancies, with only approximately 250 reported cases.^(5–7)^ Its histogenesis remains poorly understood, with hypotheses suggesting the potential origins of urothelial metaplasia or Müllerian remnants. These remnants may persist from embryonic development, originating from the Müllerian duct, which typically gives rise to the uterus, fallopian tubes, and the upper third of the vagina in females. It has been postulated that these embryonic remnants are retained in the urinary tract, contributing to the development of clear cell adenocarcinoma.^(4,5)^

Additionally, clear cell adenocarcinoma may be associated with endometriosis foci within the urinary tract or remnants of the paramesonephric ducts.^(4)^ To test the hypothesis of malignant transformation of endometriotic foci, histopathology must demonstrate endometriotic foci in association with the neoplasm.^(4,5)^

In our case, the patient presented with a primary Müllerian-type clear cell adenocarcinoma. Imaging findings ruled out a primary gynecological origin and dismissed the possibility of secondary local infiltration at a gynecological site. Given the absence of endometriotic tissues associated with the neoplasm, this suggests an alternative origin from the Müllerian duct remnants or metaplasia. This highlights the complexity of histogenesis in urethral adenocarcinomas, which is influenced by factors, such as embryonic development.

Histologically, these tumors often exhibit the characteristic features of clear cell carcinoma of the endometrium that also show a Müllerian origin, including the classic triad of tubulocystic, papillary, and diffuse patterns. They exhibited hobnail and flattened cells with abundant clear cytoplasm, moderate-to-marked nuclear pleomorphism, and frequent mitotic figures.^(4,5)^

The immunohistochemical panel in our case was positive for PAX8 and napsin A, which, when coupled with histological morphology, strongly supported the diagnosis of clear cell adenocarcinoma of Müllerian origin. This aligns with the typical immunohistochemical profile observed in Müllerian-derived cases in which PAX8 and napsin A are commonly positive, reinforcing the validity of the diagnosis. Moreover, the negativity for estrogen and progesterone receptors and GATA-3 further supports the exclusion of endometrial and urothelial origins. Additional immunohistochemical markers (HNF-1β, ARID1A, PAX2, and mismatch repair proteins) could have further corroborated the Müllerian origin;^(5,7,8)^ however, these were unavailable for testing in our setting.

Distinguishing urethral clear-cell adenocarcinoma from its morphological mimics can be challenging, particularly given its rarity and the frequent overlap in histological patterns among glandular urethral tumors. Careful evaluation of both the morphology and immunohistochemical profile is essential to reach an accurate diagnosis and exclude secondary involvement from adjacent organs or metastatic disease.^(4–6)^

Urothelial carcinoma with clear cell changes was the most common differential diagnosis. Histological findings may show clear cytoplasm and papillary architecture; however, it usually lacks the classic combination of tubulocystic, papillary, and hobnail patterns typical of Müllerian-type cell carcinoma. Immunohistochemistry plays a decisive role; urothelial carcinoma characteristically expresses GATA3 and p63/p40 and is usually negative for PAX8, which helps distinguish it from Müllerian lesions.^(4–6)^ A concise summary of the key diagnostic features is presented in table 1.

**Table 1:** Main morphological and immunohistochemical features of urethral clear cell adenocarcinoma and mimics

Entity	Main morphology	Immunoprofile	Distinguishing features / pitfalls
Müllerian-type clear cell adenocarcinoma (present case)	Tubulocystic/papillary pattern; hobnail and clear cells; high-grade nuclei	PAX8+, Napsin-A+, HNF-1β+, CK7+, CK20−, ER−, PR−, GATA3−, p63/p40−	PAX8/Napsin-A suggest Müllerian origin but are not entirely specific^(4-6)^
Urothelial carcinoma (clear cell/glandular differentiation)	Papillary or solid nests; marked atypia	GATA3+, p63+, p40+, PAX8–, Napsin-A–	May closely mimic clear cell carcinoma^(4-6)^
Nephrogenic adenoma	Small tubules, bland cytology, minimal mitoses	CK7+, PAX8–, Napsin-A–, GATA3–	Reactive/benign lesion^(4,5,9)^
Metastatic renal clear cell carcinoma	Solid/alveolar nests with delicate vasculature	CD10+, RCC+, PAX8 variable, Napsin-A–	Excluded by renal imaging and immunoprofile^(4,5)^
Metastatic gynecologic clear cell carcinoma	Similar papillary/tubulocystic pattern	PAX8+, Napsin-A+	Requires exclusion of endometrial/ovarian primary by imaging^(4,5,8)^

The clinical presentation is often nonspecific, most commonly hematuria, followed by obstructive symptoms, such as urinary retention, incontinence, or recurrent infection. The diagnosis relies on cystoscopy and imaging, and metastases from other sites must always be excluded^(5–8)^

Current management strategies for primary urethral cancer, including urothelial carcinoma, squamous cell carcinoma, and adenocarcinoma, involve a multimodal approach due to the rarity and aggressiveness of the disease. A retrospective analysis of 32 female urethral cancer patients spanning from 1997 to 2017 revealed that surgery, systemic chemotherapy, and radiation therapy were the main forms of treatment. The study identified certain risk factors associated with poorer outcomes, including tumor size ≥3cm, lymph node involvement and histological subtype, with adenocarcinoma showing the most aggressive course.^(3)^

Other studies have shown promising results with radical surgical approaches such as bladder and urethral resection, leading to complete resolution in certain cases. Overall, the heterogeneity and poor prognosis of primary urethral cancer emphasizes the importance of a multidisciplinary treatment approach encompassing surgery, chemotherapy, and radiotherapy to optimize patient outcomes.^10)^

## CONCLUSION

Here, we report a rare case of Müllerian-type clear cell adenocarcinoma of the urethra, which remains poorly understood because of its exceptional rarity. This condition should be included in the differential diagnosis of urethral tumors presenting with hematuria in middle-aged women. Accurate diagnosis depends on the recognition of its distinctive morphology and confirmatory immunohistochemistry. This study contributes to a better understanding of the regional embryologic pathways and tumorigenesis.

## Data Availability

The content is already available.
